# Relationship between Three-Dimensional Steel Fiber Statistics and Electromagnetic Shielding Effectiveness of High-Performance, Fiber-Reinforced Cementitious Composites

**DOI:** 10.3390/ma13225125

**Published:** 2020-11-13

**Authors:** Namkon Lee, Gijoon Park, Junil Pae, Juhyuk Moon, Sungwook Kim

**Affiliations:** 1 Department of Infrastructure Safety Research, Korea Institute of Civil Engineering and Building Technology, 283 Goyangdae-Ro, Ilsanseo-Gu, Goyang-Si 10223, Korea; joon7767@kict.re.kr (G.P.); swkim@kict.re.kr (S.K.); 2 Department of Civil and Environmental Engineering, Seoul National University, Seoul 08826, Korea; sdc03055@snu.ac.kr (J.P.); juhyukmoon@snu.ac.kr (J.M.)

**Keywords:** electromagnetic wave, shielding effectiveness, steel fiber distribution, high-performance fiber-reinforced cementitious composites, micro-tomography

## Abstract

This study aims to investigate the relationship between the steel fibers and the electromagnetic wave shielding effectiveness of a high-performance fiber-reinforced cementitious composite (HPFRCC). The distribution characteristics of the steel fibers and the variation of the electrical conductivity of HPFRCC as a function of the fiber content were quantified based on micro computed tomography (CT) and impedance measurements to determine their correlations with the electromagnetic shielding effectiveness. The impedance results showed that no electrical network was formed in the composite by the steel fibers and it is difficult to manufacture HPFRCC with high-electrical conductivity using steel fibers alone without CNTs or other carbon-based materials. For the steel fiber content of greater than 0.5%, the number of contact points between the steel fibers increased significantly, and the relationship between the fiber content and the number of contact points was observed. Despite the improvement of the electrical conductivity owing to the presence of the steel fibers and to the increase in the contact points between the steel fibers, the shielding effectiveness did not increase further for the steel fiber contents equal or above 1.5%. Consequently, it was found that the factor that controls the shielding effectiveness of HPFRCC is not the electrical network of the steel fibers, but the degree of the dispersion of the individual steel fibers.

## 1. Introduction

The popularity of electronic devices and their use, especially in wireless and communication systems, has resulted in problems, such as pollution attributed to electromagnetic interference (EMI) and information security violations that are (a) harmful to human health and (b) impose risks associated with the leakage of military secrets [1]. Long-term exposure to electromagnetic waves negatively affects the human body and induces the growth of tumors [2,3,4,5]. To mitigate the electromagnetic interference pollution problems, it is essential to develop electromagnetic shielding materials that act as barriers to limit the penetration of electromagnetic waves by reflection or absorption [6].

Although concrete is the most representative structural material, it is known to have extremely low electromagnetic wave shielding effectiveness compared with other shielding materials because of its low electrical conductivity (less than 1 × 10^−5^ S/cm). While existing shielding materials (copper, nickel, steel, etc.) exhibit a shielding effectiveness of approximately 60 dB or more (99.9999%), concrete materials have been reported to have a shielding effectiveness in the range of 0 to 10 dB (~90% or less). Although concrete is a nonconductor, studies are being conducted to improve its shielding effectiveness to prevent harmful effects from electromagnetic waves. To enhance the shielding effectiveness of concrete, conductive materials such as carbon nanotubes (CNTs) and steel fibers are mixed during the mixing stage, in which an appropriate amount of these materials is mixed with concrete to improve its shielding effectiveness. 

Steel fiber is the best-known material used to enhance the shielding effectiveness of concrete. In the case of cement paste that contains stainless steel fiber with a length of 6 mm and a diameter of 8 μm, the effectiveness is 58 dB at 1 GHz (sample thickness of 4.45 mm), as attained using the steel fiber at 0.90 vol.% [1]. Graphene oxide (GO) powder and conductive fibers have been investigated as fillers in the electromagnetic shielding cementitious composites. The use of both graphene oxide micro particles and short steel fibers resulted in the improvement of the EMI effectiveness of the cementitious composites. At a steel fiber content of 2.0%, a shielding effectiveness (SE) of 30 dB was measured at frequencies in the range between 1.8 and 8.0 GHz. At a steel fiber content of 2.0% and a graphene oxide content of 10%, 40 dB SE (sample thickness of 32 mm) was measured at a frequency that ranged from 1.8 to 8.0 GHz [6]. 

Carbon fiber (CF) is another material that can contribute to the enhancement of the SE. The shielding effectiveness of carbon fibers can be found in several studies. Cement paste containing 1 vol.% type B PAN-based carbon fiber (unsized) yielded a shielding effectiveness of 30 dB at 1 GHz (sample thickness of 4 mm) [7], while a 0.4% carbon fiber/cement composite yielded an SE of 19.2 dB (sample thickness of 10 mm) [8]. Carbon fiber has also been mixed with other conductive materials for the improvement of SE. The cement-based composites with 2 wt.% helical carbon fiber, 60 vol% expanded glass beads, and a thickness of 20 mm exhibits 17.8 dB of EM wave absorption performance [9]. The incorporation of Fe_3_O_4_ nanoparticles (5 wt.%) with 0.4 wt.% CF in the cementitious matrix resulted in a shielding effectiveness of 29.8 dB, which was 34.4% higher than that of the CF owing to the synergistic effect between CF and Fe_3_O_4_ nanoparticles [10]. With 0.4 wt.% GO-deposited CF/cement composites (sample thickness of 5 mm), a shielding effectiveness was 34 dB in the frequencies between 8.2 and 12.4 GHz, that is 31% higher than that of CF/cement (26 dB) [11]. 

In addition to conductive fibers, conductive powders have also been used to enhance the shielding effectiveness. Reflectivity tests using a network analyzer (frequency range: 1–18 GHz) exhibited that the cement composite produced with 25 wt.% copper slag and 6 wt.% copper powder had the electromagnetic wave absorption capacity with a 10.2 dB reflection loss and a 3.48 GHz absorption band [12]. The amount of carbon black (CB) cement-based composites (CBCC) in the percolation threshold zone were 0.36–1.34 vol.%. CBCC exhibited good performance in the absorption of electromagnetic waves. For CBCC containing 2.5 wt.% of CB, the lowest reflectivity was 20.30 dB in the frequency range of 8–26.5 GHz [13]. 

While ordinary concrete has low-shielding effectiveness because the distributed non-conductive coarse aggregates make it difficult to form a conductive network, the high-performance fiber-reinforced cementitious composite (HPFRCC) that does not contain coarse aggregates, is highly likely to have higher shielding effectiveness. The HPFRCC easily forms a conductive network because it is generally mixed with approximately 1.5–2% steel fibers and does not contain nonconductive coarse aggregates. However, it is almost impossible to find studies related to the electromagnetic wave shielding of HPFRCC. Recently, the effect of carbon nanotubes (CNTs) on mechanical properties and electromagnetic SE of ultra-high-performance concrete (UHPC) was investigated [14]. When mixed with 1.0% of CNTs, it showed a shielding effectiveness of 20 dB at a frequency of 1 GHz. In that study, the shielding effectiveness was obtained only with CNTs without using steel fibers, and it was found that the formation of the conductive pathway was well formed by setting a percolation threshold at approximately 1% of the CNT content.

The dispersion of steel fibers in HPFRCC is closely related to electromagnetic wave shielding. Micro computed tomography (micro-CT) is a powerful technology capable of obtaining images of the internal structure of a material in three dimensions (3D) nondestructively [15]. Several micro-CT-based studies have been performed on the spatial distribution and orientation of steel fibers in the cementitious matrix. Suuronen et al. [15] used X-ray microtomography to measure the spatial distribution and orientation of short steel fibers in steel fiber-reinforced concrete (SFRC). Steel fiber dispersion in SCC was investigated in [16] with the X-ray CT method. The results indicated that X-ray CT can be effectively used to determine 3D fiber dispersion. Moreover, Ruan and Poursaee [17] conducted an assessment of the fiber distribution in ultra-high-performance concrete (UHPC) using conventional imaging, CT scans, and electrical impedance tomography (EIT). The experimental results revealed that the lower flowability achieves a uniform steel fiber distribution. Miletić et al. [18] used micro-CT to investigate the orientation and distribution of steel fibers in UHPC. The results showed that the expected volume fraction obtained by the proposed method was similar to the experimental design value. The measurements of fiber orientation according to the casting procedure in UHPC were conducted in [19] using X-ray CT. The results showed that the casting method governs the orientation of steel fibers.

To the best of the authors’ knowledge, there has been limited investigation on the relationship between steel fiber distribution and electromagnetic shielding effectiveness of HPFRCC. The significance of this study is that the relationship between the mixture ratios of steel fibers and the electromagnetic wave shielding effectiveness of HPFRCC has been investigated. The distribution characteristics of the fibers and the variation of the electrical conductivity of HPFRCC as a function of the fiber content were quantified based on micro computed tomography and impedance measurements to determine their correlations with the electromagnetic shielding effectiveness.

## 2. Materials and Methods

### 2.1. Materials

The ordinary Portland cement (OPC, ASTM C150 Type I) was used in this study (Sungshin Cement Corp., Sejong, Korea). The OPC had a Blaine fineness of 3700 cm^2^/g and a specific gravity of 3.17. Fly ash and silica fume were supplied by Maxcone Corporation (Korea) and Elkem Corporation (Korea), respectively. Average grain size of silica powder used was 14 μm and a diameter of quartz sand was ranged from 100 to 800 μm. Length and diameter of steel fibers were 19.5 mm and 0.2 mm, respectively.

Table 1 lists the chemical composition of the OPC, fly ash, and silica fume used in this study. Table 2 lists the physical properties of the conductive fibers tested herein.

### 2.2. Mixture Proportions and Sample Preparation

The mixture ratio of HPFRCC is listed in Table 3. The water-to-binder weight ratio (cement + fly ash + micro silica) was 0.30. Slump flow was measured in no-hit conditions with the mini-slump flow test. The amount of superplasticizer agent (SP) was adjusted to fulfill the slump flow requirement of 180 mm. However, some of the samples could not satisfy this requirement because the slump flow did not reach 180 mm even with an increased amount of SP.

Steel fibers were added to the HPFRCC from 0.1 to 2.5 vol.% of HPFRCC. The HPFRCC was manufactured according to the study conducted by Lee et al. [20]. The fresh HPFRCC was placed in 60 × 60 × 160 mm^3^ prismatic molds and 50 mm cubic molds for alternating current (AC) impedance measurements and micro-CT scan, respectively. For the electromagnetic shielding effectiveness test, the HPFRC slurries were cast into 300 mm × 300 mm × 100 mm steel molds. The steel molds were immediately covered with polyvinyl chloride sheets to prevent surface drying. Specimen casting was conducted according to the method proposed by Yoo et al. (2017) [21].

The HPFRCC samples were stored at a temperature of 20 °C and a relative humidity (RH) > 99% in sealed conditions during first 24 h. The samples were then stored in a water bath at 90 °C for 72 h. Subsequently, the samples were oven-dried at 60 °C for 72 h to inhibit the pore solution effect on the electrical conductivity. They were then kept at 20 °C in a constant temperature and humidity chamber until the test day.

### 2.3. Test Methods

The AC impedance and electrical resistivity were measured in accordance with Lee et al. (2019) [22] and Layssi et al. (2015) [23]. LCR meters (Keysight Technologies, model: E4980A, Daejeon, Korea) were used for the AC impedance tests. Two copper electrodes (20 × 60 × 0.5 mm^3^) were embedded in the 60 × 60 × 160 mm^3^ specimen at intervals of 30 mm. The frequency was swept from 20 Hz up to 1 MHz using a logarithmic point spacing of 50 points. The AC impedance measurement was carried out with one specimen for each variable in Table 3.

**Figure 1:**
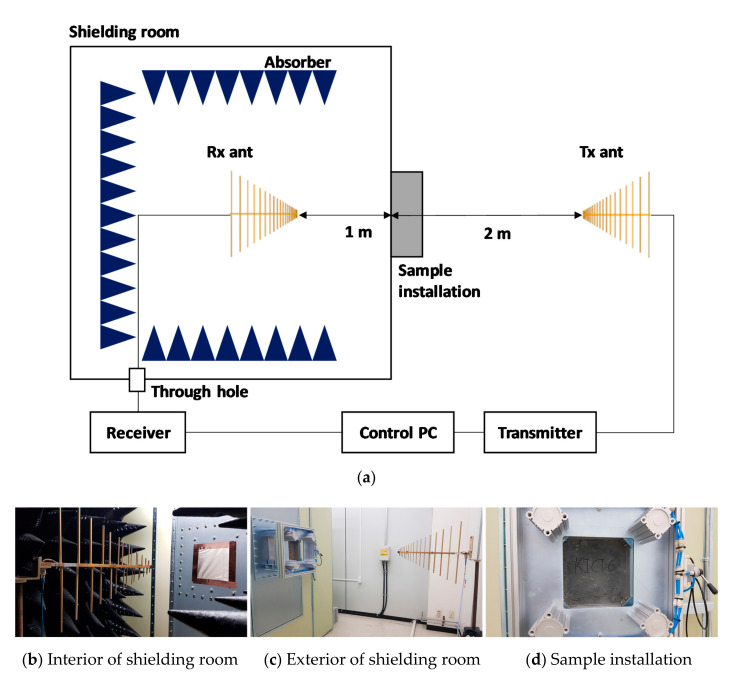
Schematic of the shielding effectiveness (SE) measurement system. (**a**) System configuration, (**b**) Receiving (Rx) antenna placed inside the shielding room, (**c**) Transmitting (Tx) antenna placed outside the shielding room, and (**d**) Sample installation.

The shielding effectiveness of the HPFRCC samples was measured according to the military standard MIL-STD-188-125 [24]. The sample thickness was 10 cm. Figure 1 shows the schematic diagram for the shielding effectiveness measurement system of the HPFRCC samples. The receiver and receiving antenna were placed in the shield room to minimize the electromagnetic waves flowing into the receiver, and the other electronic equipment and the transmitting antenna were installed outside the shield room. The two antennas employed for transmission and reception were log-periodic antennas with a measurement bandwidth of 600 MHz to 2 GHz. The distance between the antennas was 3 m, and the height was 1.2 m, as shown in Figure 1. The shielding effectiveness test was carried out with one specimen for each variable in Table 3.

Flow test was conducted according to the method suggested by Ferraris and De Larrard (1998) [25]. The unconfined compressive strength test was carried out with using a 3000 kN universal testing machine according to ASTM C39 [26], and the strength was measured with three samples for each variable in Table 3.

**Figure 2:**
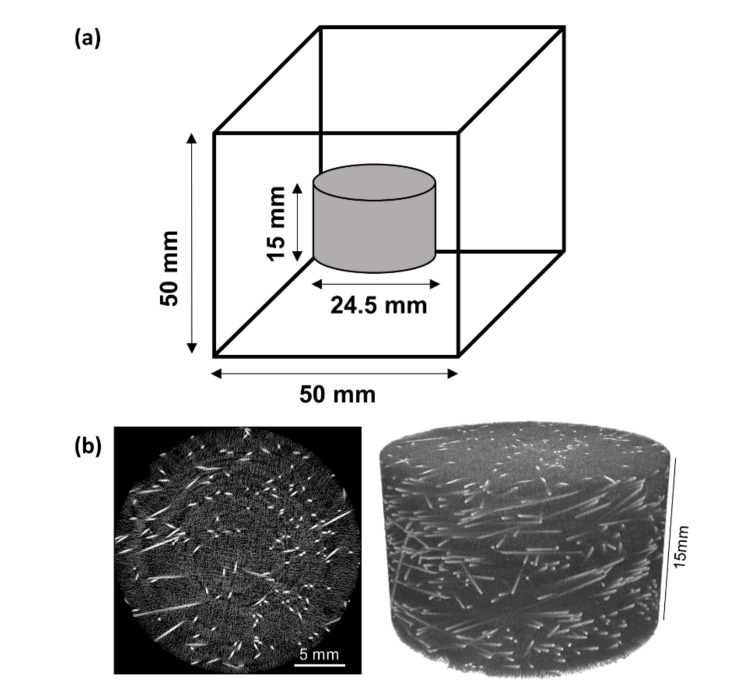
(**a**) Internal cylindrical structure obtained by micro-computed tomography (micro-CT). (**b**) Example slices (left) and three-dimensional (3D) re-conducted images (right) of specimens SF2.5.

X-ray tomographic imaging was performed with an X-ray CT scanner (Skyscan1272, Bruker, Belgium). The voltage and current of the X-ray were set to 100 kV and 100 µA, respectively. The reconstructed image acquired from the tomographic data was 2452 × 2452 pixels with a pixel resolution of 10 µm per pixel using the 2 × 2 binning mode. With an exposure time of 5000 ms and an angle step of 0.2°, 1508 layers of X-ray projection images were obtained. Considering the high density of UHPFRC, the voltage and current of the X-rays were set to the maximum to ensure ample penetration. As a result, 3D cylindrical data located at the center of the cube with a diameter of 24.52 mm and a height of 15.08 mm were obtained for each 50 mm cube (Figure 2a). Example cross-sections and 3D images acquired by micro-CT are shown in Figure 2.

There was a difficulty in the accurate segmentation of fibers from the micro-CT images. HPFRCC with high-fiber content unavoidably includes bundled fibers that should significantly affect subsequent fiber statistics, such as distribution and orientation. In view of this challenge, the following image processing steps were implemented to individually separate fibers from the original micro-CT images. First, Gaussian blur and anisotropic diffusion filters were adapted to smooth the original greyscale images while maintaining the phase boundaries of the fibers. The fibers were then segmented based on the threshold value estimated by the triangular selection algorithm [27]. The separated fibers and the matrix phases were visualized as white and black, respectively. Then, a watershed algorithm was applied to the binary segmented image to sever the bundled fibers from the individual fibers, while it maintained the phase boundaries of the fibers. Subsequently, the separated individual fibers were labeled for identification, and the information for each fiber (e.g., volumes of fibers and coordinates of the centroid and orientation) was calculated. Furthermore, the contact area between the bundled fibers could also be obtained by subtracting the segmented image after applying the watershed algorithm from the binary image. As bundled fibers were successfully separated into individual fibers, subsequent analysis of fiber statistics could be performed in a more accurate manner. The micro-CT analysis was carried out with one specimen for each variable in Table 3.

## 3. Results and Discussion

### 3.1. Flow and Compressive Strength

The experimental results of the flow and compressive strength are listed in Table 4. Except for the SF1.0 and SF2.0 samples, the compressive strength generally showed a tendency to increase as the steel fiber content increased. The SF0 sample without steel fiber yielded a slump flow of 200 mm at 1.6% of SP (superplasticizer) amount. The slump flow remained unchanged at the same level of SP up to 0.3 vol.% of steel fiber content and was decreased slightly to 190 mm at 0.4 vol.% of steel fiber content. From 0.5 vol.% of the steel fiber content, the SP content was increased to 1.8% to secure the slump flow of 200 mm. The slump flow decreased to 170 mm at a steel fiber content of 2.5 vol.% despite the SP content of 1.8%.

### 3.2. Electrical Resistivity

**Figure 3:**
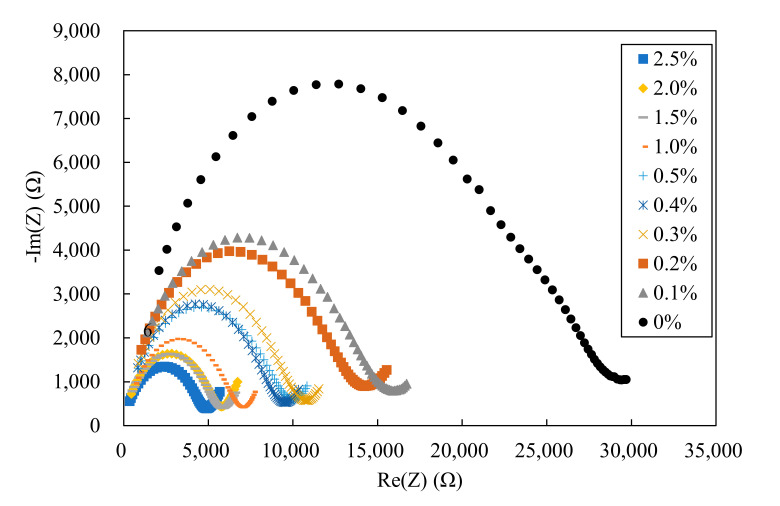
Experimental Nyquist plots for 100 MPa HPFRCC for various amounts of steel fiber.

Figure 3 shows the experimental Nyquist plots for the 100 MPa HPFRCC with different amounts of steel fiber. As the steel fiber content increases, the imaginary part of the impedance tends to decrease along with the real part impedance. It is noteworthy that the real part of the impedance decreased by approximately 1500 Ω when the steel fiber content increased from 0% to 0.1%, and it decreased by approximately 1000 Ω when the steel fiber content increased from 0.1% to 2.5%.

**Figure 4:**
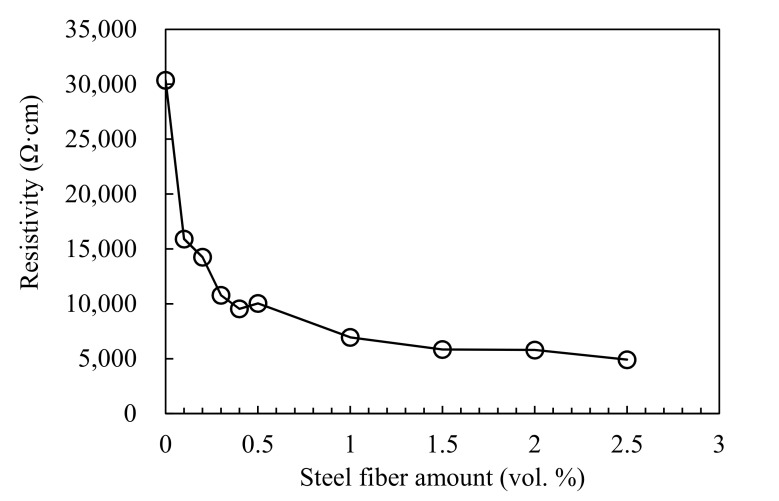
Resistivity of 100 MPa HPFRCC as a function of steel fiber.

Table 5 summarizes the resistivity of HPFRCC with various amounts of steel fiber. As reported by Wansom et al. (2006) [28], the resistivity of HPFRCC is decomposed into the composite and matrix resistance parts. The resistance of the composite and matrix is shown in Figure 3, and the results are shown in Table 5. As shown in Table 5, only the resistance of the cement matrix was measured because the SF0 sample did not contain steel fibers. For the remaining samples, the resistance of the cement composite (R_mat_) was measured while the resistance of the cement matrix (R_com_) could not be measured because the equipment had a limited frequency band (from 20 Hz to 1.0 MHz). Unfortunately, the frequency band within which it can measure the resistance of the cement matrix was from 0.01 to 20 Hz. It was found that the resistance of the composite decreased as the steel fiber content increased. As shown in Figure 4, the resistance of the composite decreased significantly up to 1.0% of the steel fiber content but decreased only slightly from 1.0% to 2.5% of the steel fiber content. The resistivity of SF2.5 sample was 5000 Ω·cm, which is much higher than the resistance of 200 Ω·cm of HPFRCC mixed with CNTs reported in a previous study [22]. This indicates that no electrical network was formed in the composite by the steel fibers. This result shows that it is difficult to manufacture HPFRCC with high-electrical conductivity using steel fibers alone without CNTs or other carbon-based materials.

### 3.3. Micro-CT, X-ray Tomography Analysis

#### 3.3.1. Fiber Distribution

**Figure 5:**
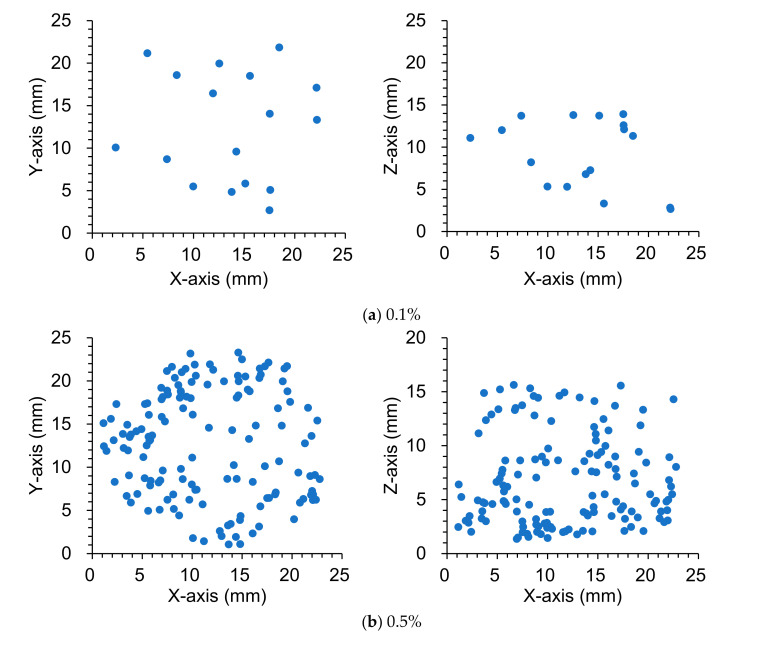

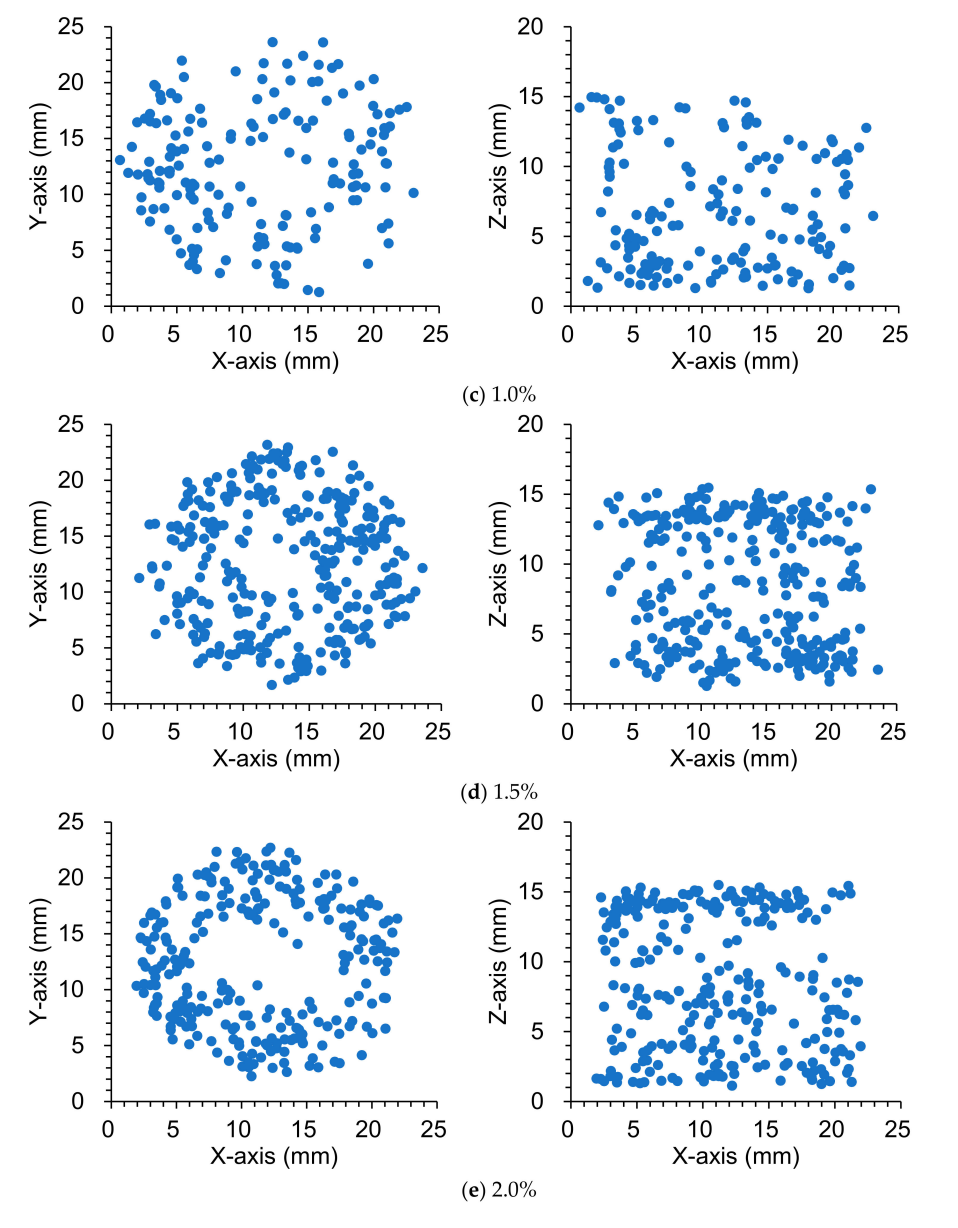

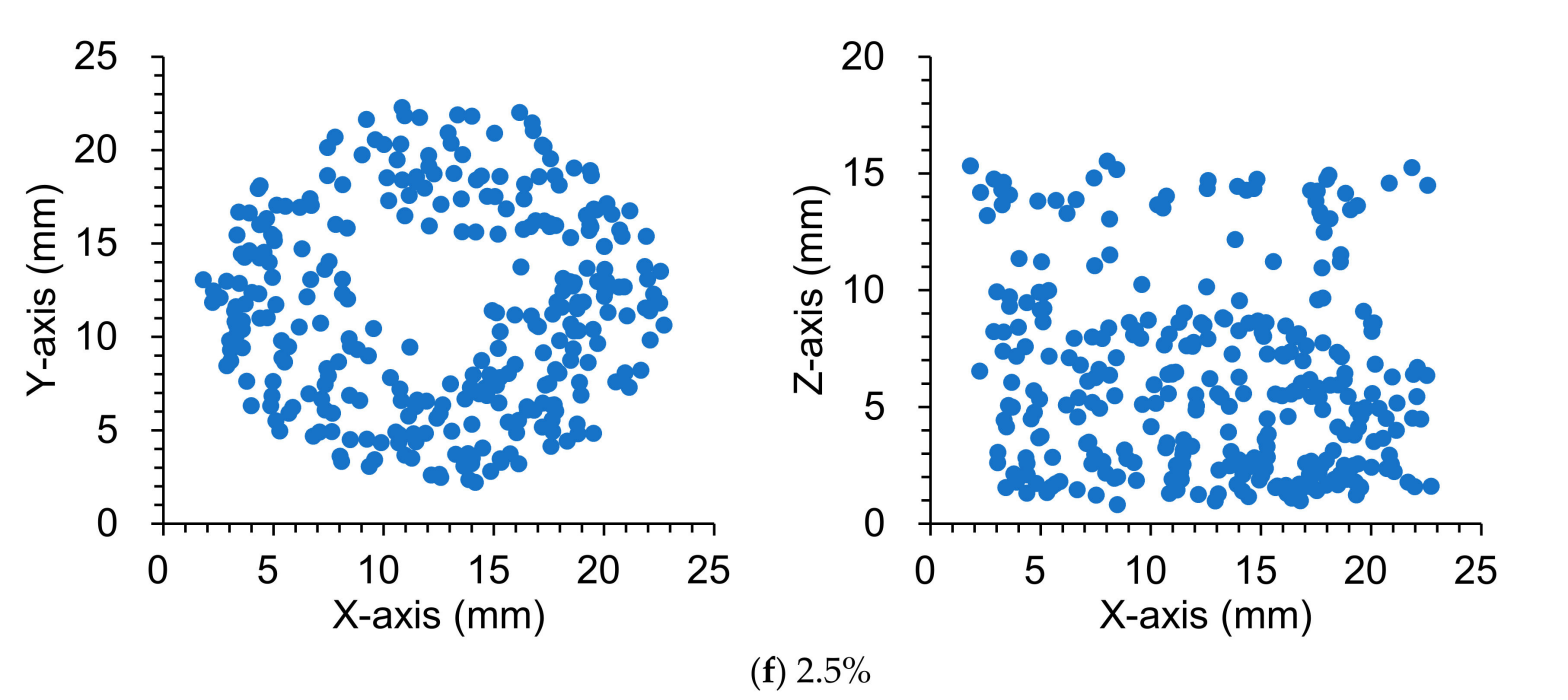
Distribution of the centroid of separate fibers in HPFRCC with various amount of steel fibers.

The distribution of the centroid of the separated fibers with various amounts of steel fiber is shown in Figure 5. The distribution of the centroid of separate fibers is represented on the xy-plane and the xz-plane. For all specimens, these distributions confirmed that the fibers were scattered uniformly throughout the sample. Meanwhile, from the results of the distribution on the xz-plane at 2.5% of steel fiber content, it was verified that a larger amount of steel fibers was distributed near the bottom of the specimen. In general, the physical separation of steel fibers occurs when the fiber content is high or the water reducing agent is excessively mixed so that HPFRCC has an excessively high fluidity. Therefore, mixing 2.5% of steel fiber content is not recommended for the stable distribution of the fiber. A notable fact is that in the distributions on xy-plane at 1.5%, 2.0%, and 2.5% of steel fiber contents, the centroid of the steel fiber is not distributed in the middle part. This is an analysis error that occurred during the separation of the bundled fiber by the proposed image processing methods, including the watershed algorithm.

#### 3.3.2. Fiber Orientation (Theta, Phi)

**Figure 6:**
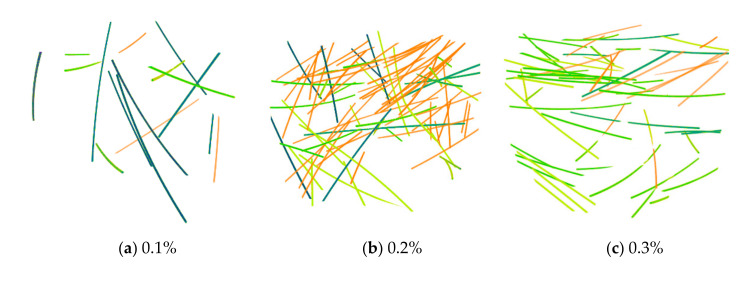

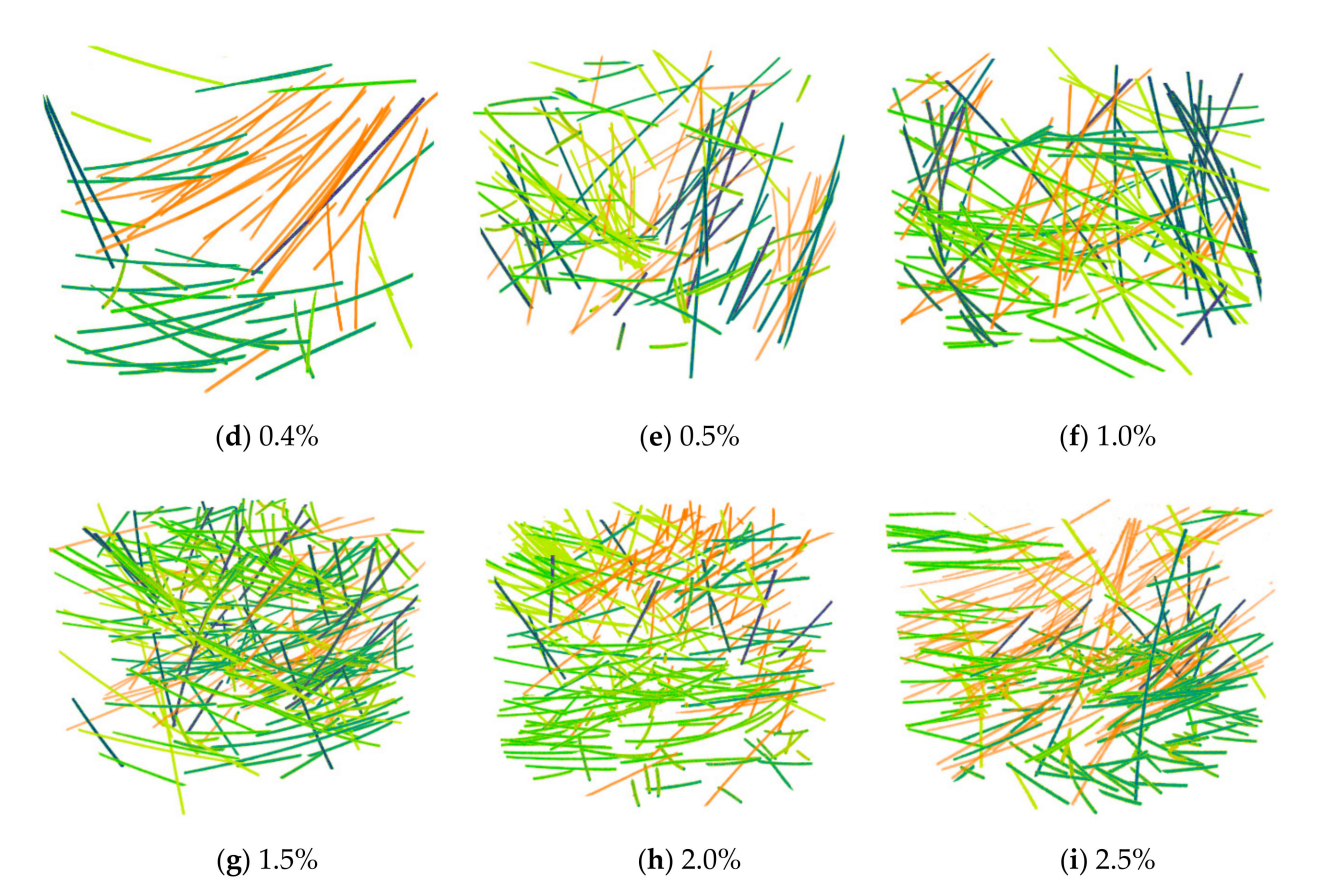
3D visualization of the orientations of individual fibers in HPFRCC. Each color represents the different 3D spherical sections cut at 90° intervals.

**Figure 7:**
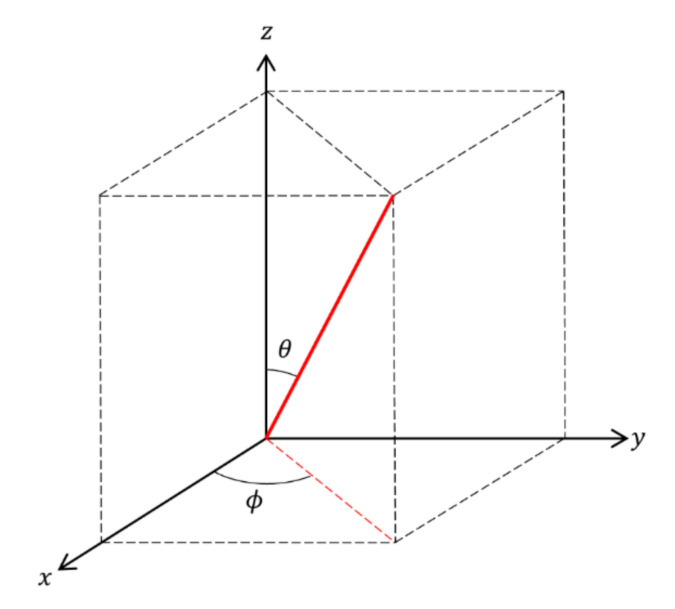
Defined orientation angles of fibers.

**Figure 8:**
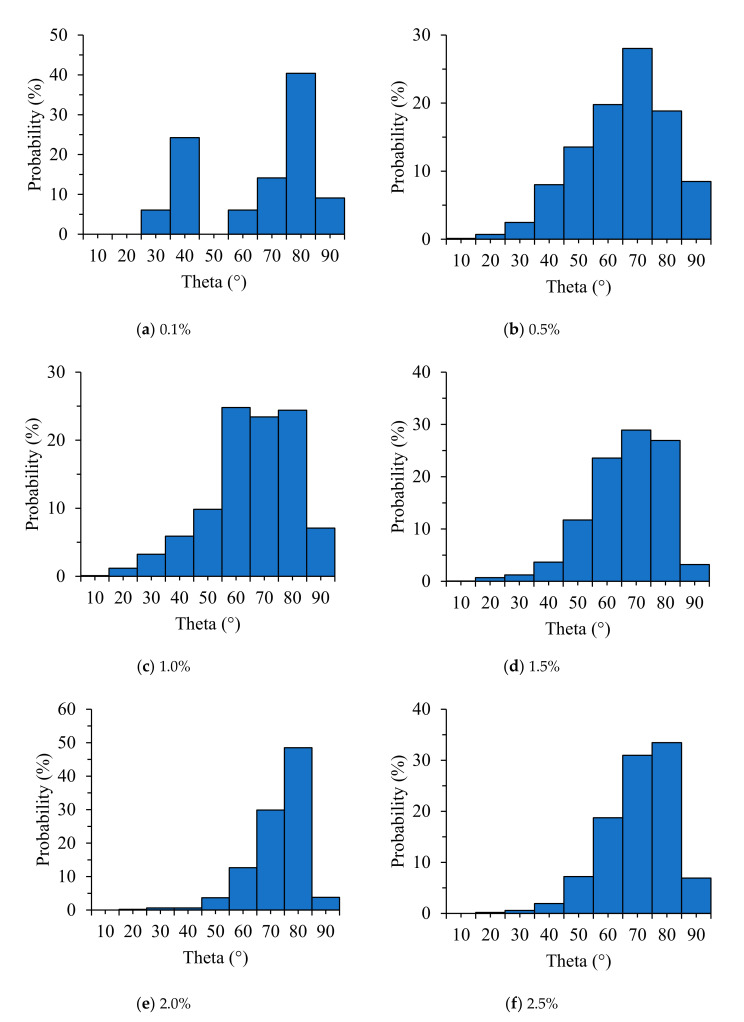
Probability density histograms of the polar angle θ for various fiber contents.

**Figure 9:**
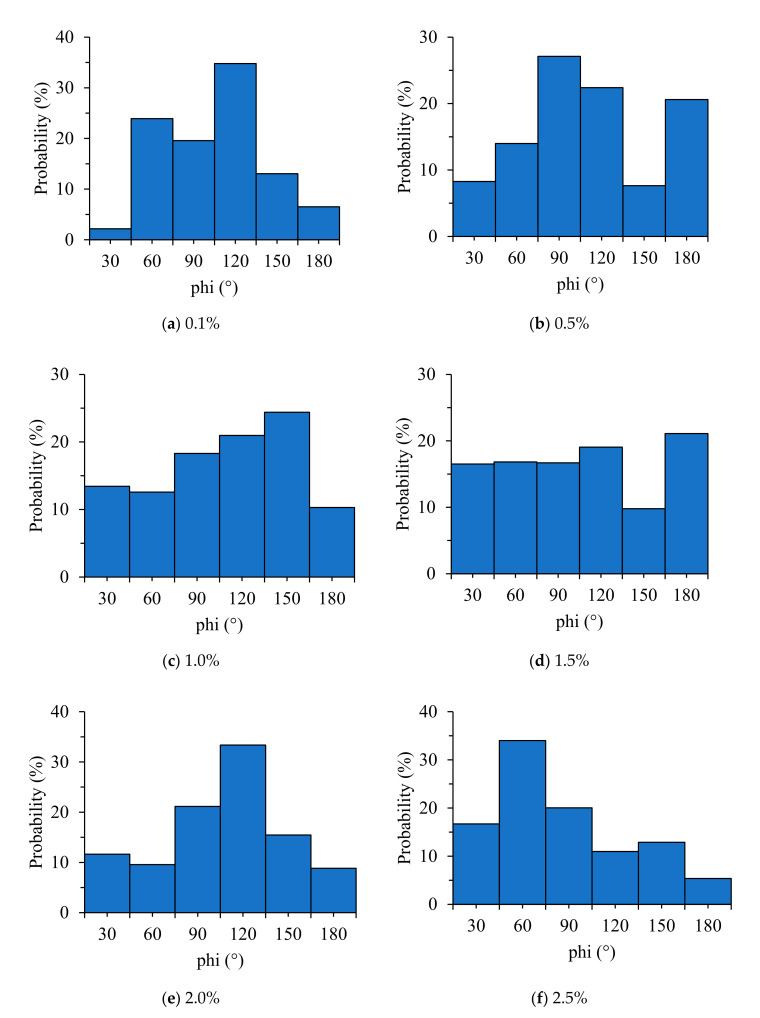
Probability density histograms of the azimuthal angle ϕ for various fiber contents.

From the proposed image processing techniques, the 3D coordination of individual fibers was investigated according to their orientation. The 3D visualizations of the fiber orientations are shown in Figure 6. Each color represents a different 3D spherical section cut at 90° intervals. It was confirmed that the steel fibers tended to be arranged almost horizontally for all specimens. This is attributed to a slight vibration applied to completely fill the mold by the material during the pouring of the HPFRCC. Subsequently, the fiber orientation was defined in the spherical coordinates to numerically examine the influence of fiber content, as shown in Figure 7. The polar angle θ is the angle between the longitudinal axis of a fiber and the positive z-axis that ranged from 0° to 90°. The azimuthal angle ϕ is the signed angle measured from the positive x-axis to the orthogonal projection of the longitudinal axis of a fiber on the xy-plane that ranged from 0° to 360°. The fiber orientation, including polar and azimuthal angles, was analyzed for each individual fiber. Probability density histograms of the polar angle θ and the azimuthal angle ϕ at various fiber contents are shown in Figure 8 and Figure 9. Most of the polar angles tend to lie in the range of 60° to 80°. This is consistent with the 3D visualization of the fiber orientations in Figure 6. The azimuthal angle ϕ does not show any trend. Considering that the pouring direction is along the z-axis, the pouring direction does not affect the fiber arrangement in the x-axis direction, and the fibers appear to be randomly distributed.

#### 3.3.3. Point of Contact between Fibers

**Figure 10:**
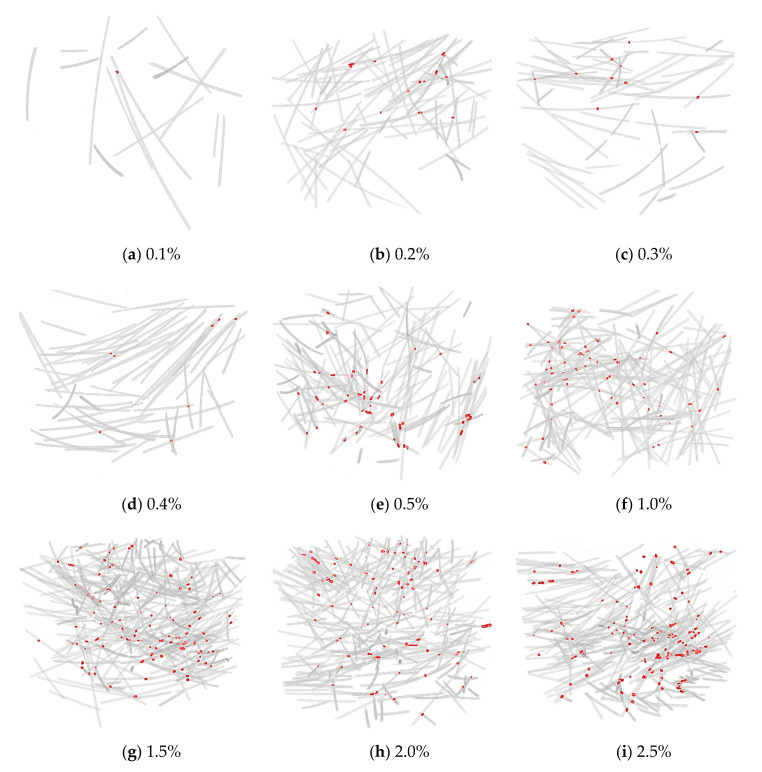
3D visualization of separated fibers and contact points. Fibers and contact region colored as blue and red, respectively.

**Figure 11:**
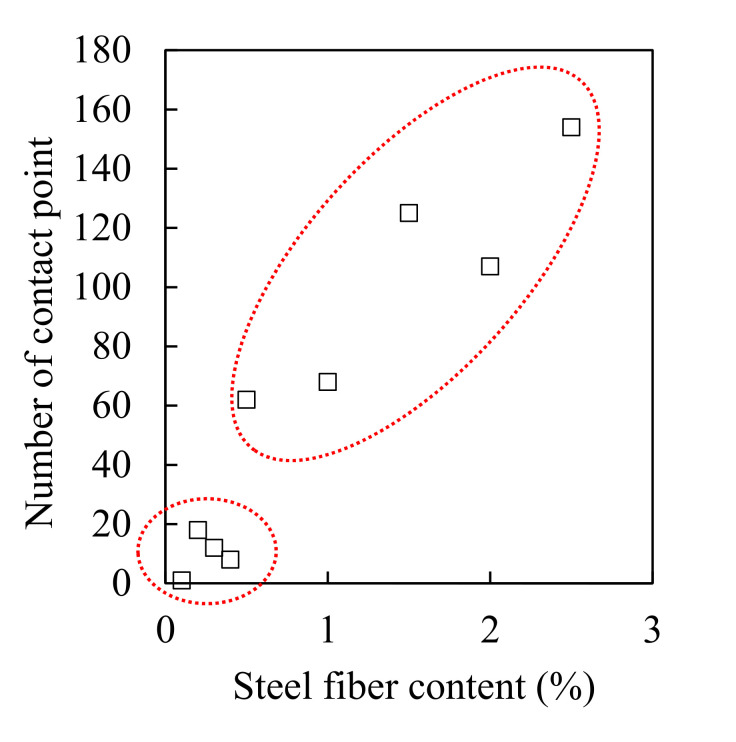
Plot of the number of contact points as a function of steel fiber content.

**Figure 12:**
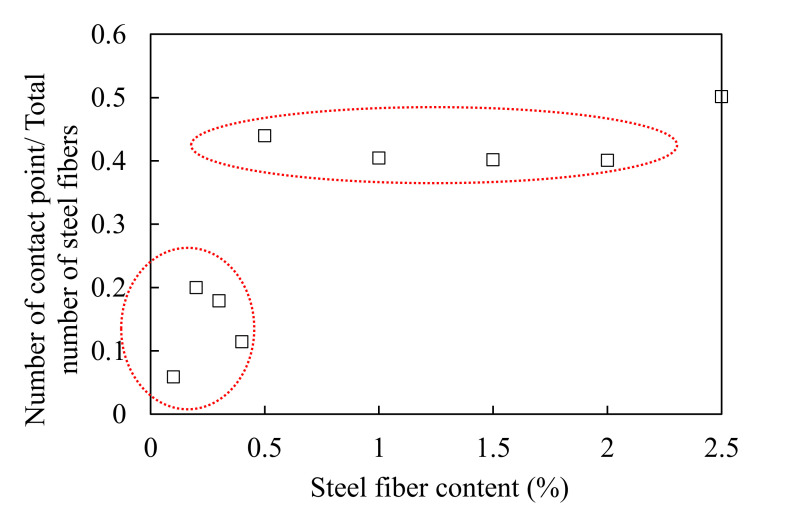
Plot of number of contact points divided by total number of steel fibers as a function of steel fiber content.

Figure 10 shows the 3D visualization of the separated fibers and contact points of all samples, and Figure 11 shows the number of contact points as a function of the steel fiber content. There is no correlation between the steel fiber content and the number of contact points up to 0.4% of the steel fiber content. This is attributed to the small amount of steel fiber and the diminutive 3D CT scan range (diameter: 25 mm, height: 15 mm). When the fiber content was greater than 0.5%, the number of contact points increased significantly, and the relationship between the fiber content and the number of contact points was observed. Figure 12 shows the value obtained by dividing the number of contact points by the total number of individual steel fibers according to the fiber content. The value was lower than 0.2% up to 0.4% of steel fiber content, and it tended to be consistent in the range of 0.4–0.45 for fiber contents >0.5%. This implies that the distribution of steel fibers is very homogeneous for fiber contents >0.5%. However, only in the case of SF2.5, this value slightly increased to 0.5 because the possibility of contact between steel fibers increased significantly due to the excessive fiber content. In the case where the range of micro-CT scanning was smaller than the size of the specimen, the fiber content should be greater than 0.5% to ensure a valid micro-CT analysis.

### 3.4. Electromagnetic SE

As shown in the shielding effectiveness measurement system in Figure 1, the side where the electromagnetic wave enters the specimen after it exits the transmitting antenna is the same as the side where the cement composite was cast into the mold. Therefore, the steel fiber mainly shows an angle of 60° to 80° from the direction in which the electromagnetic wave travels from the transmitting antenna to the receiving antenna, as shown in Figure 8. The effective area of the steel fibers capable of blocking electromagnetic waves expands when the steel fibers are arranged perpendicularly (90°) rather than in the same direction as the electromagnetic wave propagation.

The azimuthal angle of the steel fiber in the X-axis direction was strongly related to the vertical and horizontal directions of the antenna. Given that the azimuthal angle in the X-axis direction was randomly distributed, as shown in Figure 9, the effect of the vertical and horizontal directions of the antenna on the shielding effectiveness was determined to be insignificant. However, there was a slight difference in the shielding effectiveness depending on the vertical and horizontal directions of the antenna in the shielding measurement results that can be attributed to the environmental factors of the shielding room (size of the shielding room, shielding room floor, jig, etc.) rather than the arrangement of steel fibers.

**Figure 13:**
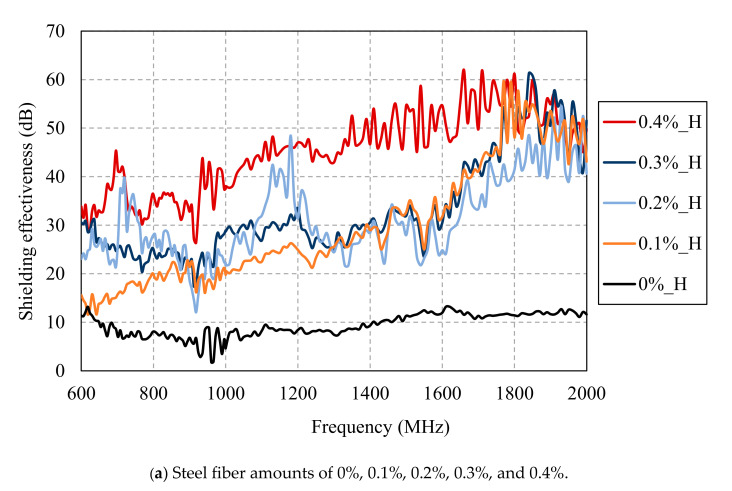

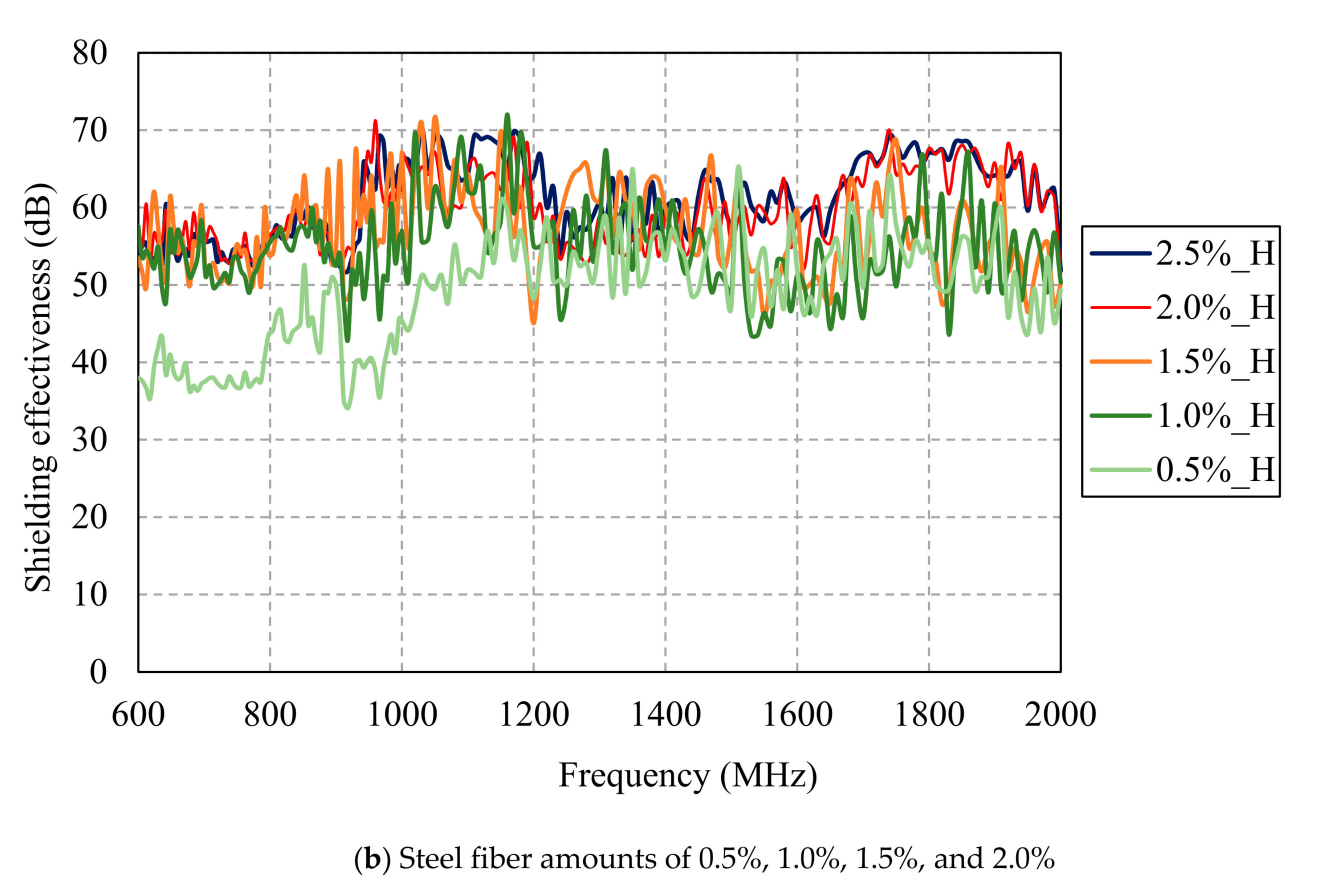
SE results of HPFRCC for different amounts of steel fibers measured with horizontal antenna.

**Figure 14:**
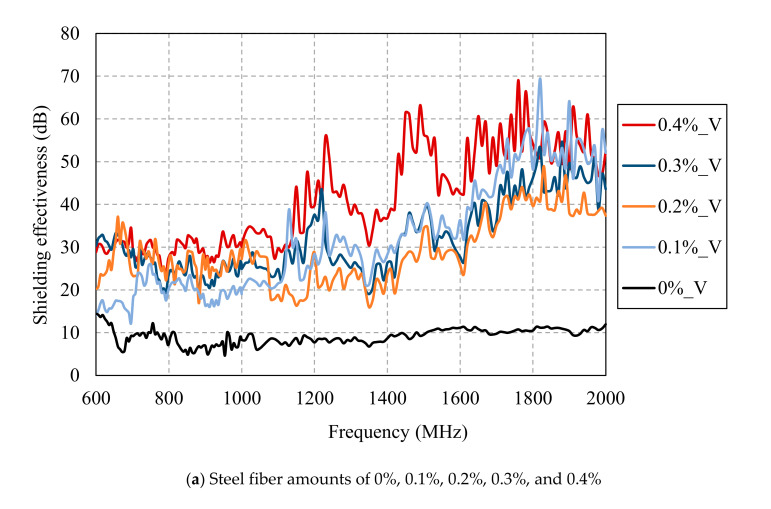

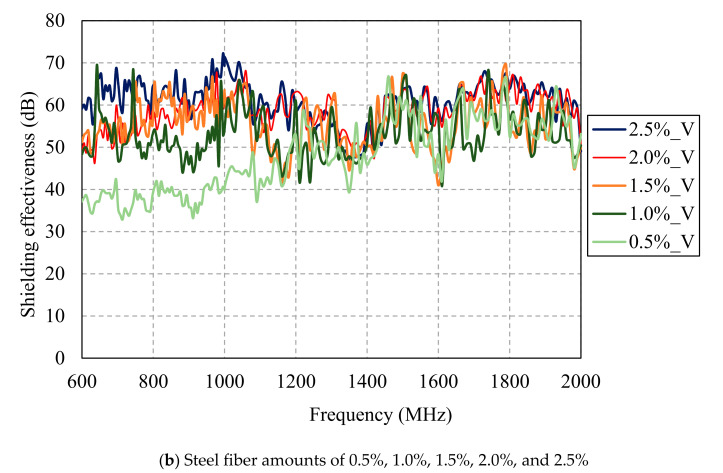
SE results of HPFRCC for different amount of steel fibers measured with a vertical antenna.

Figure 13 and Figure 14 show the SE results of HPFRCC for different amounts of steel fibers measured along the horizontal and vertical antennas, respectively. Up to the steel fiber content of 0.4%, the shielding effectiveness increased with the steel fiber content. At the steel fiber content from 0.5% to 2.5%, it increased along with the steel fiber content only at the frequencies below 1.2 GHz while it tended to remain constant regardless of the steel fiber content at the frequencies above 1.2 GHz.

**Figure 15:**
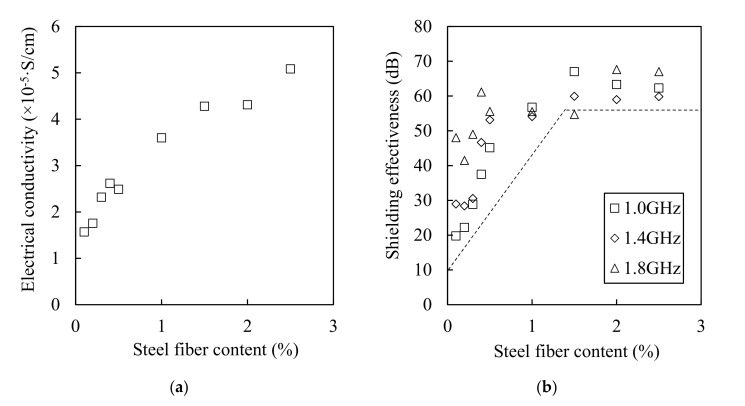
Relationships between electrical conductivity (**a**) and shielding effectiveness (**b**) as a function of steel fiber content.

**Figure 16:**
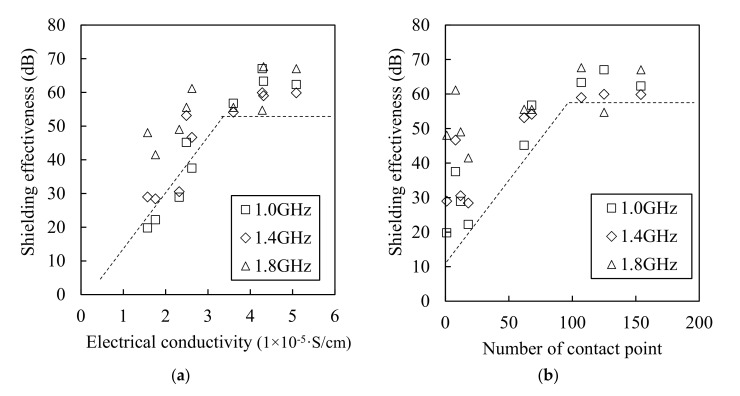
Relationship between shielding effectiveness as functions of electrical conductivity (**a**) and number of contact points (**b**).

Figure 15 shows the relationship between the steel fiber content and the electrical conductivity (a) and between the steel fiber and the shielding effectiveness (b). Although the electrical conductivity increased when the steel fiber content increased, the shielding effectiveness did not increase for contents equal or above 1.5%. The electromagnetic wave shielding effectiveness did not increase from 3 × 10^−5^ S/cm of electrical conductivity, as shown in Figure 16a, and the electromagnetic wave shielding effectiveness did not increase when the number of contact points between the steel fibers was >100 (Figure 16b). This result shows that there is a minor effect associated with the increase in the shielding effectiveness from 1.5% or more of the steel fiber content. Despite the increase in the electrical conductivity owing to the mixing of the steel fibers, and the increase in the contact point between the steel fibers, the shielding effectiveness did not increase further. This indicates that the factor that controls the shielding effectiveness of HPFRCC is not the electrical network of the steel fibers. Instead, it is closely related to the degree of the dispersion of the steel fibers. The effective area of the conductive material that can block the penetration of electromagnetic waves is important, and the effective area expands as the steel fibers are well dispersed. In other words, the most important factor in shielding electromagnetic waves is the degree of dispersion of the steel fibers. Further studies on the dispersion and electromagnetic wave shielding of steel fibers will be conducted in the future.

## 4. Concluding Remarks

This study presented experimental results and discussions to investigate the relationship between the mixture ratios of steel fibers and the electromagnetic wave shielding effectiveness of HPFRCCs. The distribution characteristics of the steel fibers and the variation of the electrical conductivity of HPFRCC as a function of the steel fiber content were quantified based on micro computed tomography (CT) and impedance measurements to determine their correlations with the electromagnetic shielding effectiveness. The following conclusions can be drawn from the results presented in this paper.

1. The real part of the impedance decreased by approximately 1500 Ω when the steel fiber content increased from 0% to 0.1%, and it decreased by approximately 1000 Ω when the steel fiber content increased from 0.1% to 2.5%. It was found that the resistance of the composite decreased significantly up to 1.0% of the steel fiber content, but it decreased only slightly from 1.0% to 2.5% of the steel fiber content. The resistivity was 5000 Ω·cm at 2.5% of the steel fiber. This indicates that no electrical network was formed in the composite by the steel fibers. This result also shows that it is difficult to manufacture HPFRCC with high-electrical conductivity using steel fibers alone without CNTs or other carbon-based materials.
2. The 3D visualizations of the fiber orientations showed that the steel fibers tend to be arranged almost horizontally for all specimens. This is attributed to a vibration work applied to completely fill the mold by the material during the pouring of the HPFRCC. Most of the polar angles, that is the angles between the longitudinal axis of a fiber and the positive z-axis, tend to lie in the range of 60° to 80°, which was confirmed by the 3D visualization of the fiber orientations. Meanwhile, the azimuthal angle ϕ does not show any trend. Considering that the pouring direction is along the z-axis, the pouring direction does not affect the fiber arrangement in the x-axis direction, and the fibers appear to be randomly distributed.
3. There is no correlation between the steel fiber content and the number of contact points between the steel fibers up to 0.4% of the steel fiber content. This is attributed to the small amount of steel fiber and the diminutive 3D CT scan range (diameter: 25 mm, height: 15 mm). Meanwhile, when the fiber content was greater than 0.5%, the number of contact points increased significantly, and the relationship between the fiber content and the number of contact points was observed.
4. The value obtained by dividing the number of contact points by the total number of individual steel fibers was lower than 0.2% up to 0.4% of steel fiber contents, and tended to be consistent in the range of 0.4–0.45 for the steel fiber contents >0.5%. This implies that the distribution of steel fibers is very homogeneous in the HPFRCCs for the steel fiber contents >0.5%. However, only in the case of SF2.5 sample, this value slightly increased to 0.5 because the possibility of contact between steel fibers increased significantly due to the excessive fiber content. In the case where the range of micro-CT scanning was smaller than the size of the specimen, the fiber content should be greater than 0.5% to ensure a valid micro-CT analysis.
5. Up to the steel fiber content of 0.4%, the shielding effectiveness increased with the steel fiber content. At the steel fiber content from 0.5% to 2.5%, it increased along with the steel fiber content only at the frequencies below 1.2 GHz while it tended to remain constant regardless of the steel fiber content at the frequencies above 1.2 GHz.
6. Although the electrical conductivity increased when the steel fiber content increased, the shielding effectiveness did not increase for the steel fiber contents equal to or above 1.5%. The electromagnetic wave shielding effectiveness did not increase when the number of contact points between the steel fibers was more than 100. Despite the improvement of the electrical conductivity owing to the presence of the steel fibers and the increase in the contact point between the steel fibers, the shielding effectiveness did not increase further. It was found that the factor which controls the shielding effectiveness of HPFRCC is not the electrical network of the steel fibers. Instead, it is closely related to the degree of the dispersion of the steel fibers. The effective area of the conductive material that can block the penetration of electromagnetic waves is important, and the effective area expands as the steel fibers are well dispersed. In other words, the most important factor in shielding electromagnetic waves is the degree of dispersion of the steel fibers. Further studies on the dispersion and electromagnetic wave shielding of steel fibers will be conducted in the future.

## Figures and Tables

**Table 1:** Chemical composition of binder materials used in this study.

Composition (wt.%)	XRF		
	OPC	Fly Ash	Silica Fume
SiO_2_	20.6	38.07	95.31
Al_2_O_3_	5.0	14.54	0.1
Fe_2_O_3_	3.4	5.42	0.35
CaO	60.7	22.78	0.21
MgO	2.6	2.67	0.8
SO_3_	2.38	5.45	0.55
K_2_O	0.98	5.83	-
Na_2_O	0.15	0.92	0.19
TiO_2_	0.27	3.62	-
P_2_O_5_	0.11	1.52	0.03
Others	<0.25	1.19	-
LOI	0.75	7.1	2.46

**Table 2:** Physical properties of steel fiber.

Type of Fiber	Density (kg/cm^3^)	Tensile Strength (MPa)	Length (mm)	Diameter (mm)
Straight steel fiber	7.8	2967	19.5	0.2

**Table 3:** Mixture ratios of high-performance fiber-reinforced cementitious composite (HPFRCC) incorporating steel fiber, carbon fiber, and milled carbon.

No.	Sample	Cement	Fly Ash	Silica Fume	Filler	Sand	w/b Ratio ^a^	SP	Steel Fiber (Vol. %) ^b^
1	Plain	1	0.2	0.1	0.2	1.2	0.30	0.015	0
2	F0.1								0.1
3	F0.2								0.2
4	F0.3								0.3
5	F0.4								0.4
6	F0.5								0.5
7	F1.0								1.0
8	F1.5								1.5
9	F2.0								2.0
10	F2.5								2.5

^a^ Binder is a mixture of cement, microsilica, and fly ash. ^b^ Volume fraction of steel fibers in HPFRCC.

**Table 4:** Flow and compressive strength of samples with various amounts of superplasticizer.

Sample	Compressive Strength (MPa)	Slump Flow (mm) (No Hit)	Superplasticizer Amount (%) by wt.% of Cement
SF0	106.5	200	1.6
SF0.1	111.1	200	1.6
SF0.5	115.3	210	1.8
SF1.0	106.6	200	1.8
SF1.5	113.6	210	1.8
SF2.0	106.4	200	1.8
SF2.5	118.0	170	1.8

**Table 5:** Resistivity of HPFRCC with various amounts of steel fiber.

Sample	Resistivity (Ω·cm)		
	Alternating Current (AC) Method		
	R_com_	Frequency (Hz) at R_com_	R_mat_ *
SF0	-	-	30,363
SF0.1	15,907	117	-
SF0.2	14,256	226.9	
SF0.3	10,776	182	
SF0.4	9544	226.9	
SF0.5	10,035	182	
SF1.0	6945	283	
SF1.5	5839	352.9	
SF2.0	5795	440.1	
SF2.5	4915	352.9	

* The frequency corresponding to R_mat_ of the plain sample was 31 Hz.
